# A global database on freshwater fish species occurrence in drainage basins

**DOI:** 10.1038/sdata.2017.141

**Published:** 2017-10-03

**Authors:** Pablo A. Tedesco, Olivier Beauchard, Rémy Bigorne, Simon Blanchet, Laëtitia Buisson, Lorenza Conti, Jean-François Cornu, Murilo S. Dias, Gaël Grenouillet, Bernard Hugueny, Céline Jézéquel, Fabien Leprieur, Sébastien Brosse, Thierry Oberdorff

**Affiliations:** 1 UMR5174 EDB (Laboratoire Evolution et Diversité Biologique), CNRS, IRD, UPS, ENFA, 118 Route de Narbonne, Université Paul Sabatier, F-31062 Toulouse, France; 2 Flanders Marine Institute (VLIZ), Wandelaarkaai 7, 8400 Oostende, Belgium; 3 Ecosystem Management Research Group, University of Antwerp, Universiteitsplein 1, 2610 Wilrijk, Belgium; 4 Station d’Ecologie Théorique et Expérimentale, UMR 5321, 09200 Moulis, France; 5 UMR 5245 EcoLab (Laboratoire Ecologie Fonctionnelle et Environnement), CNRS, INP, UPS, 118 Route de Narbonne, Université Paul Sabatier, F-31062 Toulouse, France; 6 Institut des Sciences de l’Evolution (UMR ISEM, CNRS-IRD-UM2), Université de Montpellier, 34000 Montpellier, France; 7 Departamento de Ecologia, Instituto de Ciências Biológicas, Universidade de Brasília (UnB), Campus Darcy Ribeiro, 70910-900, Brasília-DF, Brazil; 8 UMR MARBEC, (CNRS, IRD, IFREMER, UM), cc 093, Place E. Bataillon, FR-34095 Montpellier, France

**Keywords:** Freshwater ecology, Biodiversity, Biogeography, Macroecology

## Abstract

A growing interest is devoted to global-scale approaches in ecology and evolution that examine patterns and determinants of species diversity and the threats resulting from global change. These analyses obviously require global datasets of species distribution. Freshwater systems house a disproportionately high fraction of the global fish diversity considering the small proportion of the earth’s surface that they occupy, and are one of the most threatened habitats on Earth. Here we provide complete species lists for 3119 drainage basins covering more than 80% of the Earth surface using 14953 fish species inhabiting permanently or occasionally freshwater systems. The database results from an extensive survey of native and non-native freshwater fish species distribution based on 1436 published papers, books, grey literature and web-based sources. Alone or in combination with further datasets on species biological and ecological characteristics and their evolutionary history, this database represents a highly valuable source of information for further studies on freshwater macroecology, macroevolution, biogeography and conservation.

## Background & Summary

With c. 126,000 already described animal species, freshwater systems host around 10% of all animals described to date^1–3^ while occupying only 0.8% of the Earth’s surface and 0.02% of available aquatic habitable volume^4^. Among aquatic organisms, fishes are a good example of this paradox (the ‘freshwater fish paradox’ *sensu* Tedesco *et al.*^5^), with c. 40% of all described species inhabiting freshwaters, while the remaining 60% inhabiting marine habitats that comprise >99% of available aquatic habitat^6^. Besides housing a disproportionately high fraction of the global animal diversity considering the small proportion of the earth’s surface that they occupy, freshwater ecosystems are also one of the most threatened habitats on Earth^7,8^. Extinction risk for freshwater fishes, for instance, is thought to be higher than that of terrestrial organisms^9^ and recent extinction rate estimates are 112 to 855 times higher than natural extinction rates^10–12^.

Describing global scale freshwater fish diversity patterns, understanding the environmental drivers and evolutionary processes shaping such diversity and revealing the major human-related threats were the major goals that motivated the compilation of the present database. Indeed, global scale datasets allowing for biogeographical, macroecological, macroevolutionary and conservation studies were available for only a few well-documented animal taxa such as birds, mammals and amphibians^13–15^. The present database increases this list of taxa by providing occurrence data by drainage basin worldwide for the most diverse group of vertebrates (i.e. fishes), with more than 33500 species described to date (FishBase; http://www.fishbase.org), from which *c.* 40% inhabit permanently freshwater systems.

**Figure 1:**
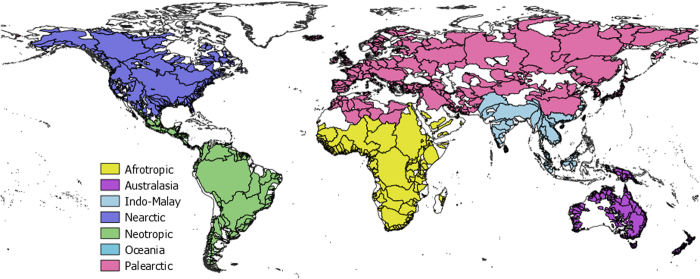
Global map indicating the drainage basins included in the database with different colours by biogeographic realm^27^. The 3119 drainage basins cover more than 80% of the Earth surface (excluding deserts), ranging from 70% for the Indo-Malay region to over 90% for the Afrotropical region.

We conducted an extensive survey of freshwater fish species distribution based on 1436 published papers, books, grey literature, databases and web-based sources, resulting in species lists for 3119 drainage basins covering more than 80% of the Earth surface (Fig. 1). Two important survey efforts were conducted, respectively completed in 2008 (ref. 16) and 2013 (ref. 17). To date, these databases have been used in several studies that have increased our understanding of freshwater fish species distributions. These studies allowed to accurately map global patterns of native^18^, endemic^19^ and non-native^20^ freshwater fish species richness and to reveal their environmental and human-related determinants. The databases were also used to evaluate non-native species influence on native communities structure^21^, to forecast climate change effects on species extinction processes^11^ and to analyse effects of current and future scenarios of species introductions on fish faunas homogenization processes^22–25^. Recent studies also focused on analysing the influence of past river connections on the present distribution of native fish species^17^, on analysing geographical and trait-based differences in diversification rates and origin of actinopterygian fish families^5^, and on evaluating human-related extinction drivers^12^.

Although the database has already provided a lot of insightful knowledge, it still represents a valuable source of information for further studies on freshwater macroecology, macroevolution, biogeography and conservation. For instance, the present dataset could serve to identify diversity hotspots and to generate a global map of ichthyogeographic regions by combining data on the distributions and phylogenetic relationships of species, allowing *in fine* the identification of geographic areas harbouring distinct evolutionary histories. Furthermore, in association with data on the time and place of origin of species or on species functional traits, the global occurrence dataset could provide new insights on the macroevolution of freshwater fishes or approach the functional characteristics of communities. Those forthcoming approaches would surely help designing large scale conservation priorities for freshwater fishes.

The database is organised in three sub-datasets and one shapefile. The first dataset contains the species occurrence records by drainage basin along with their native or non-native status and the corresponding FishBase species code and valid name. The second dataset, which is simply the export of the shapefile attributes table, contains geographic information on the drainage basins (e.g. geographic coordinates, surface area). The third dataset contains the list of references that were used to build the species lists for each of the drainage basins. This reference list is obviously not definitive and updates of the database will be performed regularly to include new occurrence records, the distribution of newly described species, species lists of new drainage basins and nomenclature changes in the always moving taxonomy.

## Methods

### Information sources

This global database of freshwater fish species distribution results from a joint collaboration between three French research institutes, i.e. the University Paul Sabatier in Toulouse (UPS), the National Museum of Natural History (MNHN) and the Research Institute for Development (IRD). The financial support necessary to build this database mainly came from two projects: the ‘Freshwater Fish Diversity’ (National Agency for Research: ANR-06-BDIV-010) and ‘BioFresh’ (7th Framework European program, Contract N°226874) projects. Starting in 2003, we conducted an extensive survey of literature published from 1960 to 2014 on native and non-native freshwater fish species at the drainage basin grain. This survey was complemented with web-based sources from national and international biodiversity inventory initiatives compiling either or both collection and field sampling data.

Our efforts were mainly devoted to find information sources providing complete fish species lists of a given drainage basin, except for some large basins (e.g. the Amazon basin) where we cumulated sub-drainage basin species lists and point sampling locations to obtain the most complete possible coverage of the entire drainage basin. We also used local or regional check lists such as local inventories of stream reaches or inventories based solely on a given family or genus to complement our species lists and for cross-checking available information at the drainage basin scale. The resulting database was gathered from 1436 sources including published papers, books, grey literature and web-based sources that included museum collections, national or regional initiatives compiling monitoring data (mainly for developed countries), continental scale atlases of species distribution and international biodiversity initiatives. When published information was found in languages not handled by any of the team members (e.g. national inventory reports or books), a translator kindly helped us to ensure the collection of correct information on river basins, species lists and location of the species.

### Species, taxonomy and status

Sub-species were not considered due to limited data availability and all occurrences not identified to species level were discarded (i.e. occurrences giving only genus names commonly abbreviated to sp., species *affinis* commonly abbreviated to: sp. aff., aff., or affin. or species *confer* abbreviated to cf.). Species migrating between both marine and freshwater environments where systematically included in the database. Concerning marine and estuarine species occasionally occurring in freshwaters, these species may be reported in the database but their distribution information should not be considered as exhaustive in any case, as these systems are not the focus of the database.

All species scientific names are reported in the database as given in each information source. These species names were then carefully checked for typing errors and misspellings. Because taxonomy is a ‘moving target’, species names were standardized based on valid species names and their synonyms reported in FishBase using the ‘rfishbase’ package^26^ from the R environment (http://www.R-project.org). For those species names that did not match with any synonym or valid name from FishBase, a manual search was applied in the Catalogue of Fishes (http://researcharchive.calacademy.org/research/ichthyology/catalog/fishcatmain.asp). This last step allowed finding valid species names and species recently described that are still not included in FishBase. For recently described species not yet validated by FishBase or species only considered valid by the Catalogue of Fishes, a temporary code was created starting by ‘x’ (0.04% of all valid species). The remaining species names were considered as invalid and excluded from the database (only 0.6% of all species names). The final standardized species list has 14953 valid names avoiding biases due to synonyms and uncertain identifications (see ‘Technical Validation’). According to FishBase, from this list of 14953 valid names our database contains the distribution of 13721 species inhabiting fresh or brackish waters, the remaining being marine species also entering fresh or brackish waters, but not recorded as such by FishBase. As a whole, the database harbours 101779 occurrence records (i.e. single species-drainage basin couples).

A native or exotic status was assigned to each species occurrence record based on the information provided by the sources and further checked (see ‘Technical Validation’). An exotic species is defined as a directly or indirectly (e.g. via artificial channels) introduced species that established in the considered drainage basin. Exotic species included in the database are supposed to complete all their life cycle in the considered basins and to present self-sustaining populations in those basins. When an exotic species occurrence was acknowledged to be unsuccessful (i.e. failure of establishment of that species in the drainage basin) or needing regular release of new individuals to maintain the presence of the introduced species (i.e. stocking), the species was not included in the basin’s species list. Species that might be globally extinct or extirpated from a drainage basin were considered as native in the database because the inventory of freshwater fish diversity loss was not targeted (see for instance Dias *et al.*^12^ for a compilation of extirpated freshwater fish species for Western Europe and North America).

### Drainage basin location and names

Each drainage basin was assigned a unique name that can be used as an identifier and was characterized by its location in one of the eight terrestrial biogeographic realms (as described by Olson *et al.*^27^; Fig. 1), the country (or main country for shared drainage basins), its endorheic or exorheic type of water flow, its geographic coordinates at the river mouth (for exorheic drainage basins), the geographic coordinates of its centroid and its drainage surface area.

A specific geographic referential (Fig. 1) was built by modifying the 30 sec HydroSheds layer^28^ to improve the delimitation and accuracy of drainage basins. For instance, some small coastal drainage basins were included in one single HydroSheds polygon but were considered as separate basins because having distinct outlets to the sea. Some drainage basins from oceanic islands have no HydroSheds code simply because not considered in the HydroSheds shapefile. Maps and geographic information available in the compiled literature and web-based sources were used to locate, name and improve our drainage basins layer, complemented by country and continental scale geographic data (e.g. Faunafri project for the African continent; http://www.poissons-afrique.ird.fr/faunafri/) and local topographic maps. This new geographic referential is provided as a shapefile to facilitate future uses of the database.

### Updates and limitations

Species are continuously being discovered and freshwater fishes are no exception, even in well-know regions^29^. Rivers are also continuously being explored and re-explored by freshwater scientists. The database is obviously not complete and definitive, and we aim to support the database with regular updates, ideally with bi-annual steps, depending on the resources and funding. Three main factors will be considered in future updates: (1) new or previously non available data sources with species lists or records for additional drainage basins or drainage basins already present in the database; (2) distribution of newly described species; and (3) nomenclature changes in the taxonomic classification. The technical validation procedures described below will also be applied to any new information included in the database. Researchers having access to new data that want this information to be included in the database can send the references or data to the corresponding author PAT. This information will be included, after validation, in the next update release. The resulting new versions of the database will be released through *Figshare* and also through the more specialized *Freshwater Biodiversity Data Portal* (http://data.freshwaterbiodiversity.eu/) to ensure the long-term availability of the database.

All biogeographic realms are well represented in terms of surface coverage (Fig. 1). There are however some regional gaps that will be gradually filled in the next updates of the database. For instance, Indonesian islands, coastal rivers of Peru and Northeast Brazil are regions where only few drainage basins are informed in the database. In these regions the scarce existing information is not easily available. Southeast Asia is the less well represented region in terms of surface coverage (Fig. 1), which is certainly related to the low number of freshwater taxonomists working in this highly diverse region^30^. All these spatial gaps in the database will be prioritized in future updates through literature and web-based sources monitoring.

## Data Records

The database is organised in three datasets and one shapefile: the species occurrence records, the drainage basins and the information sources table. The three tables are in csv format (columns separated by comas) and the shapefile in ArcGis shp format (Data Citation 1). The drainage basins table is given in.csv and shapefile formats. Both formats can be linked to the species occurrence table using the unique drainage basin names to visualize and analyse species distribution using any adapted software (e.g. R or QGIS, http://qgis.osgeo.org).

- The species occurrence records table has six columns: (1) the name of the drainage basin, (2) the scientific name of the fish species according to the information source, (3) the native or exotic status of the occurrence records, (4) the taxonomic serial number (TSN) from the Integrated Taxonomic Information System (ITIS, https://www.itis.gov/) when available, (5) the FishBase code when available, (6) the FishBase or Catalogue of Fishes valid scientific name at the time of releasing the database, (7) the occurrence status which can be either ‘valid’ or ‘questionable’ (see Technical Validation).
- The geographic information on drainage basins is organised in nine columns given in table and shapefile formats: (1) the unique drainage basin name, (2) the main country where it belongs, (3) the corresponding biogeographic region, (4) the endorheic or exorheic status, (5) and (6) the longitude and latitude coordinates of the drainage basin outlet to the sea (only for exorheic drainages), (7) and (8) the centroid longitude and latitude coordinates of the drainage basin, (9) the surface area of the drainage basin.
- The information sources table has three columns: (1) the drainage basin names, (2) the type of information sources (e.g. published paper, book, report, online database, PhD Thesis), (3) the references used to build the freshwater fish species list for the corresponding drainage basin.

## Technical Validation

### Taxonomic validation

Each species name found in a given information source was confronted to the valid and synonym species names lists from FishBase and the Catalogue of Fishes to ensure the validity of the identifications provided in the information source. After taxonomic validation, 103 invalid (unknown) species names were excluded from the database.

### Species distribution and status validation

Occurrence records were carefully reviewed by the database’ contributors. When several information sources were used to compile the species list of a given drainage basin, particular attention was given to cross check the occurrence records and ensure a good spatial representation of the drainage basin to avoid (or at least minimize) incomplete species lists. Because occurrence data available in FishBase is often incomplete, FishBase was only used as a secondary source to collect species distribution data and to check that the resulting species distributions from our database corresponded to the broad information given in FishBase.

Because only a few (mostly migratory) freshwater species can occur in more than one biogeographic realm, the distribution of every species occurring in more than one realm was carefully verified. Similarly, for species distributed in a single realm, when one or more occurrences were inconsistent with the actual known distribution of a species (i.e. the presence in a drainage located far away from a group of drainages where the species is known to occur), these occurrences were qualified as ‘questionable’. Particular care was taken with the occurrences of all species considered exotic at least in one drainage basin. The native and exotic distributions of those species were carefully checked to avoid any status error.

## Additional information

**How to cite this article**: Tedesco, P. A. *et al.* A global database on freshwater fish species occurrence in drainage basins. *Sci. Data* 4:170141 doi: 10.1038/sdata.2017.141 (2017).

**Publisher’s note: ** Springer Nature remains neutral with regard to jurisdictional claims in published maps and institutional affiliations.

## Supplementary Material
